# The microbiome structure of decomposing plant leaves in soil depends on plant species, soil pore sizes, and soil moisture content

**DOI:** 10.3389/fmicb.2023.1172862

**Published:** 2023-08-14

**Authors:** Gian Maria Niccolò Benucci, Ehsan R. Toosi, Fan Yang, Terence L. Marsh, Gregory M. Bonito, Alexandra Kravchenko

**Affiliations:** ^1^Plant Soil and Microbial Sciences, Michigan State University, East Lansing, MI, United States; ^2^Great Lakes Bioenergy Research Center, Michigan State University, East Lansing, MI, United States; ^3^Microbiology and Molecular Genetics, Michigan State University, East Lansing, MI, United States

**Keywords:** soil microbiome, CONSTAX2, metabarcoding, ITS rDNA, 16S rDNA, detritusphere, soil pores, leaf litter

## Abstract

Microbial communities are known as the primary decomposers of all the carbon accumulated in the soil. However, how important soil structure and its conventional or organic management, moisture content, and how different plant species impact this process are less understood. To answer these questions, we generated a soil microcosm with decomposing corn and soy leaves, as well as soil adjacent to the leaves, and compared it to control samples. We then used high-throughput amplicon sequencing of the ITS and 16S rDNA regions to characterize these microbiomes. Leaf microbiomes were the least diverse and the most even in terms of OTU richness and abundance compared to near soil and far soil, especially in their bacterial component. Microbial composition was significantly and primarily affected by niche (leaves vs. soil) but also by soil management type and plant species in the fungal microbiome, while moisture content and pore sizes were more important drivers for the bacterial communities. The pore size effect was significantly dependent on moisture content, but only in the organic management type. Overall, our results refine our understanding of the decomposition of carbon residues in the soil and the factors that influence it, which are key for environmental sustainability and for evaluating changes in ecosystem functions.

## Introduction

Adding aboveground plant residues to the topsoil can increase soil fertility, improve hydraulic properties, enhance carbon sequestration, and reduce erosion (Miguez and Bollero, 2005; Scholberg et al., 2010). Sustainable agriculture management practices that involve residue additions include cover cropping, green manure cropping, and crop residue incorporation by tillage (Lal, 1997). Such practices are growing in popularity worldwide and are particularly important in organically based agriculture and in agricultural systems in developing countries. The benefits of incorporating plant residues stem from their decomposition within the soil, which provides soil carbon and nutrient inputs and is driven by microorganisms (Lehtinen et al., 2014; Liu et al., 2017).

Micro-environmental conditions within the soil matrix influence microbiome activity and composition (Chenu et al., 2001; Mummey and Stahl, 2004; Wolf et al., 2013). Soil pores are known to play a major role in shaping soil micro-environments (Kravchenko and Guber, 2017). They enable gas and liquid transport, impact microbial colonization of the soil matrix (Dechesne et al., 2003; Long and Or, 2009; Wang et al., 2013), and create physical barriers between microbial communities (Treves et al., 2003) that can either reduce or enhance accessibility to predators (Wright et al., 1995) and other stress factors (Harvey et al., 2021). Connectivity among the soil micro-environments, facilitated through liquid bridges, is a major driver of the diversity of microbial communities within the soil matrix (Tiedje et al., 2001; Long and Or, 2005; Carson et al., 2010). Accounting for the characteristics of the soil pore space in numerical modeling is necessary for understanding the mechanisms and drivers of microbial dynamics and activity (Golparvar et al., 2021). However, while significant attention has been given to the role of pore characteristics in influencing microorganisms in bulk soil, defined as the soil not affected by plant residues or live plant roots (Bickel and Or, 2020; Nunan et al., 2020; Xia et al., 2022), relatively less is known about how such characteristics contribute to microbiome dynamics around incorporated plant residues.

The soil in the immediate vicinity of decomposing plant residue is known as the detritusphere (Kögel-Knabner et al., 2023), and physical properties in this zone drive the rate of residue decomposition and the fate of decomposition products (Kravchenko et al., 2017; Kim et al., 2020). A greater abundance of large pores in the detritusphere stimulates decomposition and leads to greater quantities of residual carbon being fully decomposed into CO_2_ and emitted into the atmosphere (Toosi et al., 2017). The prevalence of small pores stimulates the diffusion of decomposition products into the surrounding soil matrix, enriching it with new C inputs but also potentially stimulating microbial activity, thus priming the loss of native soil organic matter (Toosi et al., 2017).

In this study, we used microcosms to test the impact that soil pore size, moisture, and plant tissue quality have on fungal and bacterial dynamics and the incorporation of leaf litter residue into soil across space and time. We hypothesized that the decomposing residue itself would drive microbial community composition changes in the soil and that environmental conditions within the soil matrix, specifically the presence and size distribution of soil pores and the level of soil moisture, would define the composition of microbial communities on the decomposing residue and in the surrounding detritusphere. Assessment of the microbial community composition over a time course, i.e., at 7, 14, and 24 days, improves our ability to detect diversity patterns that with only one sampling time would not be possible to detect. It also provides insight into longer-term trends and factors involved in microbial turnover. We explored their role in microcosm systems from soils of contrasting long-term agricultural management histories, namely, conventional and organic row crop agriculture practices, and with residues (leaves) of two plant species common in conventional row crop agriculture, namely, corn [*Zea mays* (L.)] and soybean [*Glycine max* (L.)].

## Materials and methods

### Study design

A detailed description of the study site and the setup of the microcosm experiment is provided by Toosi et al. (2017); thus, here we only briefly highlight the key components of the experiment. The soil for the microcosms was collected from two contrasting agricultural management practices, namely, conventionally fertilized corn-soybean-wheat rotation (Conv) and biologically based corn-soybean-wheat rotation with winter cover crops (Bio), implemented since 1989 at the Long-Term Ecological Research site at Kellogg Biological Station, Michigan. During each 3-year rotation cycle (the Bio practice), the cover crop red clover (*Trifolium pratense* L.) is frost-seeded into winter wheat and then incorporated into the soil 10 months later prior to corn planting, and the cereal rye (*Secale cereale* L.) is planted after corn harvest and incorporated prior to soybean planting. The studied soil is Kalamazoo loam (fine-loamy, mixed, and mesic Typic Hapludalf) (Robertson and Hamilton, 2015).

The soil material dominated by large pores, referred to further on as the large pore soil, consisted of a 1–2 mm aggregate fraction obtained by sieving air-dried bulk soil. The soil material dominated by small pores, referred to further on as small pore soil, was created from a subset of the 1–2 mm fraction by crushing and sieving the soil to a 0.05–0.1 mm size range. Creating small pore material from the large-pore material in this study ensured maximum consistency between the inherent chemical and biological properties of the two materials; however, we are aware that the procedure could have potentially affected soil microorganisms (Powlson, 1980). X-ray-computed microtomography of the soil materials revealed that the large pore material had a substantial presence of >30 μm Ø pores, which represented the pore space in-between the 1 and 2 mm aggregates, and of < 2 μm Ø pores from within the aggregates. The pores space of the small pore material was dominated by 5–10 μm Ø pores, with no >30 μm Ø pores present (Toosi et al., 2017). The microcosms were constructed so as to maintain the same bulk density of 1.1 g cm^−3^, so both materials had the same 58% total porosity.

The treatment design for the incubation experiment consisted of the following factors: two agricultural management practices (Conv and Bio), two soil materials with contrasting pore size diameters (PSD: large and small pore materials), two soil moisture levels (18 and 28% volumetric water contents), two plant residue substrates (corn and soybean leaves), and no residue treatment (control). Since the colonization of a new substrate by soil microbiota is dynamic and therefore changes with time, we sampled the microcosms at three time points (7, 14, and 24 days after the start of the incubation). Three replicated microcosms were prepared for each treatment combination, for a total of 216 microcosms. Samples were processed as three experimental blocks in a randomized complete block design.

Each microcosm was 8 mm in diameter and 10 mm in length (Supplementary Figure S1) and contained a Ø7 mm dry leaf disk placed in-between two equal soil layers (0.45 g above and 0.45 g below the leaf). Microcosms were incubated at 20°C in the dark. At each sampling time, the microcosms were randomly assigned to the specific sampling time point, taken out of the incubation, and prepared for microbial analyses. Each control microcosm (without a plant leaf) was processed as a single sample. From each treatment microcosm with plant leaf, we procured three samples for microbial analysis, representing what we consider to be three ecological niches differing in quantity and quality of the nutrient sources available for the microorganisms. These consisted of the remains of the plant leaf itself, the soil layer at a 0–2 mm distance from the leaf, and the soil layer at a 3–5 mm distance from the leaf. The latter two samples are referred to as soil adjacent (near soil) to the leaf and soil non-adjacent (far soil) to the leaf, respectively. The samples were placed on ice immediately after cutting and then kept frozen at −80°C until further analysis. In addition, baseline microbial analyses were conducted in air-dry samples of large- and small-pore starting materials from conventional (T1) and organic (T4) managements that were not subjected to incubation.

This experiment was a component of a larger study that examined the effects of management practices, PSDs, soil moisture level, and plant leaf source (corn vs. soybean) on leaf decomposition, the emission of CO_2_ and N_2_O during incubations, the distribution of leaf decomposition products within the soil, and soil priming effects. The findings on these other components of the study have been published elsewhere (Kravchenko and Guber, 2017; Toosi et al., 2017) and thus provide auxiliary information for analyzing the data from the experiment described here.

### DNA extraction, library preparation, and sequencing

DNA was extracted from soil samples with the MoBio Power Soil kit according to the vendor's protocol, with the exception that a Biospec Mini-Beadbeater-16 was used for cell disruption. Approximately 0.25–0.5 g of soil was extracted for each sample. Samples were shaken for 1.5 min at 25°C. DNA yield was quantitated with a Nanodrop Spectrophotometer. Total soil DNA was amplified and sequenced at the Michigan State University sequencing core facility. Briefly, to assess fungal communities, the ITS region was amplified using the primer sets ITS1F12 (5′-GAACCWGCGGARGGATCA) and ITS2 (5′-GCTGCGTTCTTCATCGATGC). Amplification products were run in the same manner as the V4 amplification products (below) but on a separate MiSeq v2 flow cell.

To assess prokaryote communities, the microbial 16S rRNA gene V4 regions were amplified using primer sets 515F (5′-GTGCCAGCMGCCGCGGTAA-3′) and 806R (5′-GGACTACHVGGGTWTCTAAT-3′) following the method described by Kozich et al. (2013). Amplicons of 16S rRNA gene V4 regions were pooled and run on a standard MiSeq v2 flow cell with a 500-cycle reagent kit (PE250). Base calling was done using the Illumina Real-Time Analysis (RTA) version 1.18.54, and the output of RTA was demultiplexed and converted to FastQ format using the Illumina Bcl2fastq version 1.8.4.

### Fungal and prokaryotic sequence processing

Raw forward and reverse Illumina ITS reads were quality evaluated with FastQC (Andrews, 2010) and merged with PEAR (Zhang et al., 2014). Primers and adapters were removed with Cutadapt (Martin, 2011). Reads were quality filtered (Edgar and Flyvbjerg, 2015; Edgar, 2016), de-replicated, removed from singleton sequences, and clustered into operational taxonomic units (OTUs) based on 97% similarity using the UPARSE algorithm (Edgar, 2013). Taxonomy assignments were performed in CONSTAX2 (Liber et al., 2021) using the UNITE sequence database (Kõljalg et al., 2013).

Raw forward and reverse Illumina 16S reads were processed as previously described (Rieke et al., 2018) with the following modifications. Briefly, we used Ribosomal Database Project (RDP) Paired-end Reads Assembler (Cole et al., 2014) to merge the primer-trimmed pair-ended reads to 250–280 bases and a minimal Q score of 25. Using BLAST, we confirmed that the assembled 16S rRNA gene V4 sequences shorter than 250 bases or longer than 280 bases were non-microbial. Vsearch (2.4.3, 64-bit) (Rognes et al., 2016) was used to remove chimeras *de novo*, followed by removing chimeras by reference using RDP 16S rRNA gene training set sequences (No. 15). High-quality and chimera-free sequences were then clustered at 97% sequence similarity by CD-HIT (4.6.1) (Fu et al., 2012). The taxonomy of each representative OTU sequence was identified using the RDP Classifier (Wang et al., 2007; Fu et al., 2012) with a confidence cutoff of 50% (-c 0.5). Finally, OTUs detected fewer than five times across all samples were removed.

### Statistical modeling

For each marker gene (i.e., ITS and 16S), otu_table (McDonald et al., 2012), taxonomic classifications, representative OTU sequences, and metadata files were imported into the R statistical environment (R Core Team., 2023) and combined with the *phyloseq* package (McMurdie and Holmes, 2014). To standardize the sequencing depth across all samples, we rarefied all samples to the minimum sample size (i.e., 1,010 sequences for the fungi and 13,377 sequences for the prokaryotes) in the *phyloseq* R package (McMurdie and Holmes, 2013).

To explore differences in microbial community beta-diversity, we analyzed two components, namely, (i) community structure, defined as the difference in multivariate space between samples and sample groups and (ii) community dispersion, defined as multivariate variance within each sample group. Community structure was investigated using principal coordinate analysis (PCoA) of the Bray-Curtis distance matrix with the function “ordinate” in *phyloseq* (McMurdie and Holmes, 2014). A permutational multivariate analysis of variance (Permanova) was used to test differences among *a priori* defined sample groups (Anderson, 2001) with the function “adonis” in the *vegan* R package (Oksanen et al., 2019). To assess the amount of multivariate dispersions (Anderson et al., 2006) around centroids, we used the “betadisper” function in *vegan*. Statistical differences in dispersion were assessed through pairwise permutational ANOVA, using the “anova” function in the *car* R package, with 9,999 permutations. All *P*-values were corrected based on the Benjamini-Hochberg method (Benjamini and Hochberg, 1995).

To explore which bacterial genera will follow the decomposing residue vs. soil and the increasing vs. decreasing time trends, we first conducted a 3-way factorial ANOVA for the abundances of individual OTUs. To identify the leaf-dominating and soil-dominating genera, the ANOVA was followed by contrasts comparing the leaf with near soil and the leaf with far soil and a non-incubated control, tested simultaneously (*P* < 0.01). Then, the abundances of genera identified as either leaf- or soil-dominating were subjected to linear regression with time as the independent variable to identify those that exhibited a clear positive or negative linear trend (*P* < 0.05).

Alpha diversity, OTU richness, and Shannon diversity indexes were calculated in *vegan* with the “specnumber” and “diversity” functions of the *vegan* package (Oksanen et al., 2019). The Shannon index was standardized to 0–1 to allow for easier comparisons across groups, as previously explained (Benucci et al., 2022). Significant differences (*P* ≤ 0.05) in alpha diversity were assessed by a Wilcoxon test, with P-values corrected with the Benjamini-Hochberg method (Benjamini and Hochberg, 1995). All graphs were plotted in the *ggplot2* (Wickham, 2016) and *ggpubr* (Kassambara, 2020) R packages. Minimal graphical adjustments to improve the figures' visibility were performed in *Inkscape* (Inkscape Project, 2020).

## Results

### Sequencing results

This study resulted from community data from 252 samples that yielded 2,193,913 (8,671.6 ± 6,448.2 mean reads and standard deviation per sample, respectively) ITS reads and 3,701,268 (14,629.52 ± 7,687.7) 16S reads in the otu_table after quality filtering. The data were rarefied at 1,010 reads per sample for ITS and 13,377 for 16S.

### Beta diversity

**Figure 1:**
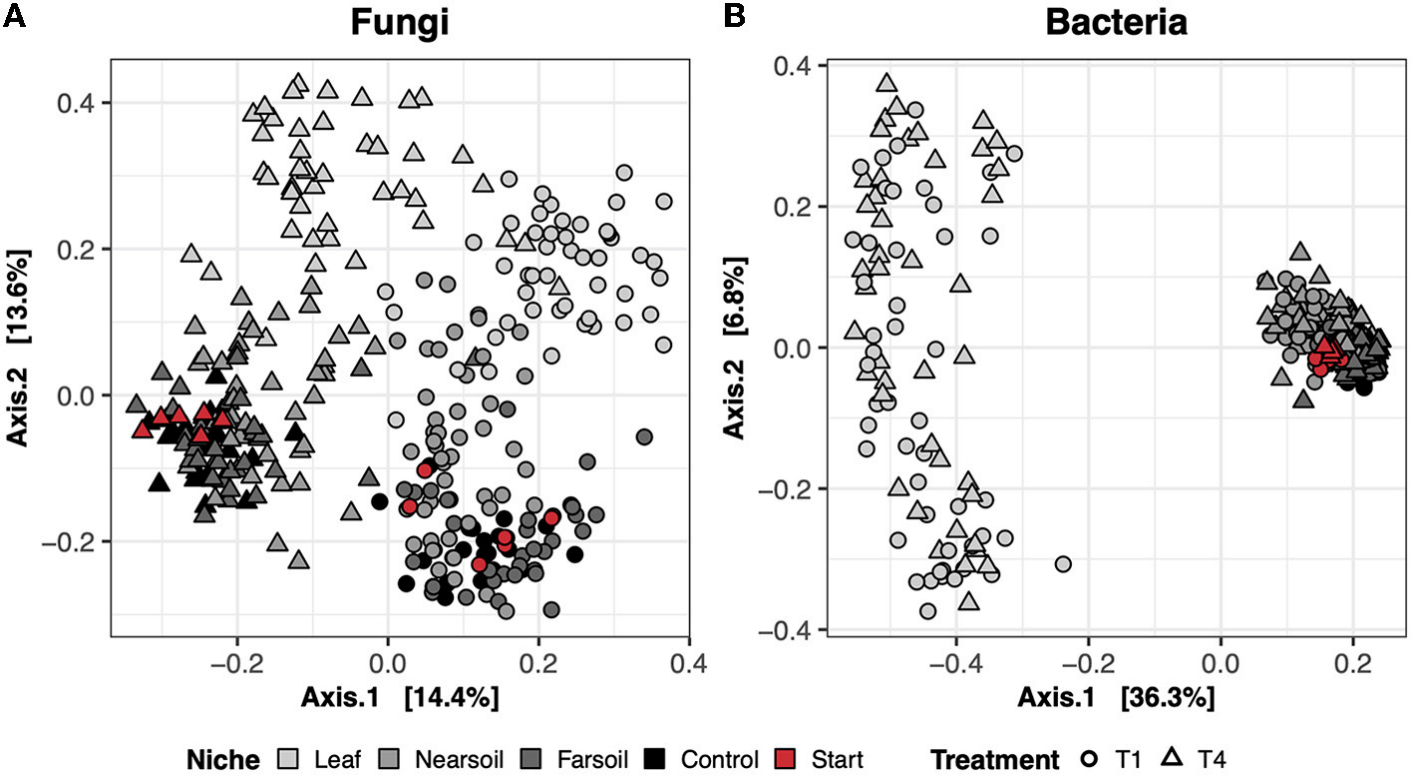
Principal Coordinate Analysis (PCoA) based on Bray-Curtis dissimilarity matrices of fungal **(A)** and bacterial **(B)** communities. Incubated samples that did not contain leaves are referred to as control (black), and dry samples of the soil materials used in the study (prior to incubation) are referred to as start (red).

In the dry control samples (i.e., the soil materials used in the study tested prior to incubation), the long-term history of contrasting agricultural management practices (T1 = conventional vs. T4 = organic) influenced both fungal and bacterial microbiomes (Supplementary Table S1). However, neither fungal nor bacterial communities differed between the large- and small-pore soil materials (Supplementary Table S1). In the study samples, the largest amount of variation in composition was present across different niches (i.e., leaf, near soil, and far soil), as hypothesized, which accounted for roughly 16% and 39% of the total variance in fungal and bacterial communities, respectively (Supplementary Table S1). Differences across niches and treatments are clear in the principal coordinate analysis (PCoA) ordination plots based on the Bray-Curtis dissimilarity of fungal (Figure 1A) and bacterial communities (Figure 1B), with clear clustering of samples along the first and second PCoA axes, respectively. Fungal communities were also more clearly impacted by soil treatment (T1 = conventional vs. T4 = organic), which explains nearly 11% of the variance, and, to a lesser extent, by plant species (i.e., corn or soy), pore size (i.e., small or large), and moisture content (i.e., low or high). Bacterial communities were also impacted by pore size (~2.5%), moisture content (~2%), treatment (~2%), and plant species (~1.5%), but these effects were hidden by the effect of niche. Significant interactions (*P* ≤ 0.05, after Benjamini-Hochberg *P*-value correction), mainly involving niche and other factors, were also present, but, in general, the amount of variation explained was negligible (Supplementary Table S2).

**Figure 2:**
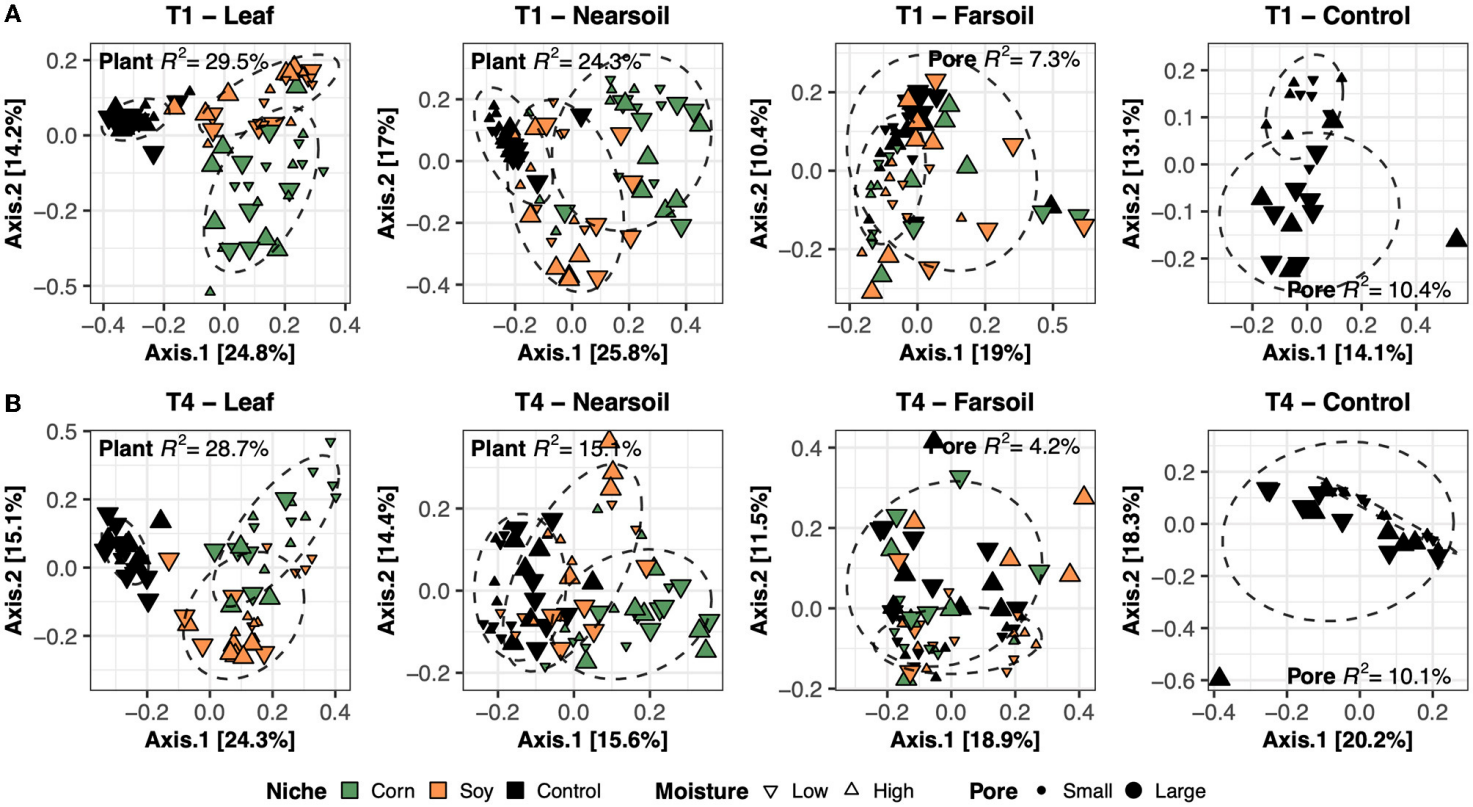
Principal Coordinate Analysis (PCoA) based on Bray-Curtis dissimilarity matrices of fungal microbiomes. PCoA of all samples grouped by treatment and niche: T1-leaf, T1-near soil, T1-far soil, and T1-control **(A)** and T4-leaf, T4-near soil, T4-far soil, and T4-control **(B)**. Sample points are coded according to plant (color), moisture (shape), and size (pore). Factors that explained the most variation in the data are reported, as are the *R*^2^ and 75% confidence level ellipses assuming a normal distribution.

**Figure 3:**
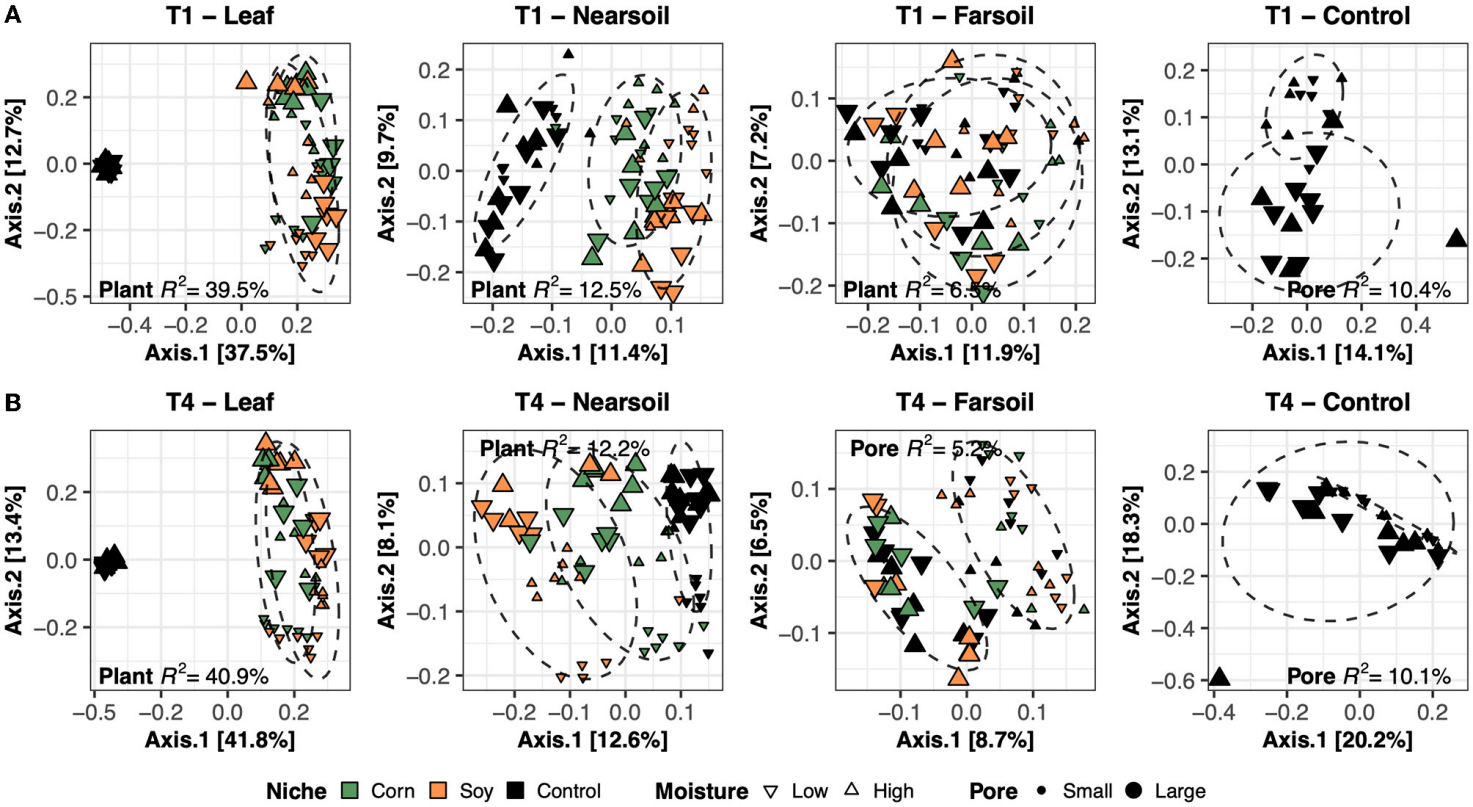
Principal Coordinate Analysis (PCoA) based on Bray-Curtis dissimilarity matrices of bacterial microbiomes. PCoA of all samples grouped by treatment and niche: T1-leaf, T1-near soil, T1-far soil, and T1-control **(A)** and T4-leaf, T4-near soil, T4-far soil, and T4-control **(B)**. Sample points are coded according to plant (color), moisture (shape), and size (Pore). Factors that explained the most variation in the data are reported, as are the *R*^2^, and 75% confidence level ellipses assuming a normal distribution.

To better evaluate the impacts of all the variables, the datasets were divided by niche and treatment into eight subsets composed of leaf-T1, leaf-T4, near soil-T1, near soil-T4, far soil-T1, far soil-T4, control-T1, and control-T4. The PCoA ordinations generated for each subset showed significantly different clusters of samples for both fungi (Figure 2) and bacteria (Figure 3), as supported by the Permanova tests.

Overall, the Permanova [P ≤ 0.05, after Benjamini-Hochberg (BH) correction] results showed that plant species and pore size were the two main drivers of the communities in both T1 and T4 and for each studied niche. In particular, plant species were always the major driver of variation in the leaf and the near soil niches (and also in the far soil for the bacterial microbiome in T1), while pore sizes impacted the far soil (i.e., soil further away from the leaf) niches; control samples were impacted by pore sizes the most (Table 1). Microbiome variance attributed to plant species ranged from about 29% in the T1-leaf to 15% in the T4-near soil of the fungal microbiome and from about 40% in the T1-leaf to 5.1% in the T4-far soil of the bacterial microbiomes, with no substantial difference between T1 and T4. Microbiome variance attributed to pore sizes ranging from about 7.3% in T1-far soil (10.5% in the T1-control samples) to 3.1% in T1-leaf of the fungal microbiome and from about 8.9% in T4-leaf (8.8% in the T4-control samples) to 5% in T1-leaf of the bacterial microbiome, with, in general, higher variance in T4 compared to T1 if we do not consider the control samples. Moisture content was most important in shaping the bacterial rather than fungal microbiome. Significant effects were present in T1-far soil (4.1%), T4-leaf (3.9%), and T4-near soil (3.2%) in the fungal microbiomes, and T1-leaf (8.4%), T1-near soil (3.3%), T4-leaf (5.3%), T4-near soil (3.6%), T4-far soil (2.7%), and T4-control samples (7.2%) in the bacterial microbiomes.

**Table 1:** Permanova (Permutational Multivariate Analysis of Variance Using Distance Matrices) and Betadisper (Multivariate Homogeneity of Groups Dispersions) models on the subsetted fungal and bacterial datasets according to slice and treatment (leaf-T1, leaf-T4, near soil-T1, T1-control, near soil-T4, far soil-T1, far soil-T4, and T4-control).

***Fungi***	***Permanova***						***Betadisper***		
**Group**	**Factor**	**Df**	**Sum.Sq**	**F**	**R** ^2^	**P.adj**	**Sum.Sq**	**F**	**P.adj**
T1-Leaf	***Plant***	2	4.22612	14.90845	0.29459	**0.00080**	0.0132	0.5592	0.5743
T1-Leaf	*Pore*	1	0.44086	3.11042	0.03073	**0.01840**	0.0883	13.6263	**0.0004**
T1-Nearsoil	***Plant***	2	2.85516	11.13978	0.24329	**0.00080**	0.0233	1.7466	0.1824
T1-Nearsoil	*Pore*	1	0.44797	3.49560	0.03817	**0.00880**	0.0425	14.7884	**0.0003**
T1-Farsoil	***Pore***	1	0.74554	4.85338	0.07261	**0.00080**	0.3237	71.3412	**0.0000**
T1-Farsoil	*Moisture*	1	0.42277	2.75218	0.04118	**0.00800**	0.0063	0.5691	0.4537
T1-Control	***Pore***	1	0.32534	2.38360	0.10468	**0.00040**	0.0427	10.4906	**0.0041**
T4-Leaf	***Plant***	2	4.03977	15.91940	0.28679	**0.00080**	0.0507	2.0021	0.1436
T4-Leaf	*Pore*	1	0.92249	7.27047	0.06549	**0.00080**	0.0299	4.4552	**0.0387**
T4-Leaf	*Time*	2	0.81393	3.20743	0.05778	**0.00080**	0.0202	1.2462	0.2946
T4-Leaf	*Moisture*	1	0.55793	4.39724	0.03961	**0.00160**	0.0067	0.8304	0.3656
T4-Leaf	*Pore:Moisture*	1	0.37736	2.97408	0.02679	**0.02000**	-	-	-
T4-Nearsoil	***Plant***	2	1.60929	6.19018	0.15103	**0.00080**	0.0094	0.5129	0.6012
T4-Nearsoil	*Pore*	1	0.40427	3.11009	0.03794	**0.00160**	0.2103	36.0006	**0.0000**
T4-Nearsoil	*Moisture*	1	0.34383	2.64510	0.03227	**0.01120**	0.0066	0.7250	0.3976
T4-Farsoil	***Pore***	1	0.35941	2.63748	0.04163	**0.01440**	0.3542	56.6875	**0.0000**
T4-Farsoil	*Plant:Pore:Moisture*	1	0.40467	2.96966	0.04687	**0.00480**	-	-	-
T4-Control	***Pore***	1	0.30512	2.49281	0.10162	**0.01240**	0.1199	18.9037	**0.0003**
***Bacteria***									
**Group**	**Factor**	**Df**	**Sum.Sq**	**F**	**R** ^2^	**P.adj**	**Sum.Sq**	**F**	**P.adj**
T1-Leaf	***Plant***	2	7.99222	33.13792	0.39641	**0.00080**	0.2459	32.4638	**0.0000**
T1-Leaf	*Moisture*	1	1.69730	14.07491	0.08418	**0.00080**	0.0029	0.4915	0.4857
T1-Leaf	*Time*	2	1.22462	5.07759	0.06074	**0.00080**	0.0127	1.4587	0.2398
T1-Leaf	*Pore*	1	1.01912	8.45110	0.05055	**0.00080**	0.0002	0.0480	0.8273
T1-Nearsoil	***Plant***	2	0.96853	5.43286	0.12532	**0.00080**	0.0010	0.6545	0.5231
T1-Nearsoil	*Time*	2	0.53755	3.01534	0.06956	**0.00080**	0.0003	0.1776	0.8377
T1-Nearsoil	*Pore*	1	0.45416	5.09507	0.05877	**0.00080**	0.0018	2.0833	0.1536
T1-Nearsoil	*Moisture*	1	0.25621	2.87432	0.03315	**0.00080**	0.0012	1.7753	0.1873
T1-Farsoil	*Time*	2	0.52353	2.90084	0.08460	**0.00080**	0.0000	0.0285	0.9719
T1-Farsoil	***Plant***	2	0.40441	2.24080	0.06535	**0.00080**	0.0013	0.9940	0.3765
T1-Farsoil	*Pore*	1	0.36626	4.05886	0.05919	**0.00080**	0.0032	5.2212	**0.0261**
T1-Control	*Time*	2	0.36715	2.03374	0.16551	**0.00040**	0.0220	2.1863	0.1398
T1-Control	***Pore***	1	0.19382	2.14727	0.08737	**0.00960**	0.0427	10.4906	**0.0041**
T4-Leaf	***Plant***	2	7.65287	37.25758	0.40881	**0.00080**	0.2337	20.6704	**0.0000**
T4-Leaf	*Pore*	1	1.67765	16.33514	0.08962	**0.00080**	0.0187	3.0142	0.0873
T4-Leaf	*Time*	2	1.57711	7.67806	0.08425	**0.00080**	0.0400	5.0084	**0.0095**
T4-Leaf	*Moisture*	1	0.98904	9.63021	0.05283	**0.00080**	0.0010	0.3096	0.5799
T4-Leaf	*Pore:Moisture*	1	0.52480	5.10997	0.02803	**0.00720**	-	-	-
T4-Nearsoil	***Plant***	2	0.88452	5.07130	0.12185	**0.00080**	0.0012	0.7689	0.4678
T4-Nearsoil	*Pore*	1	0.39821	4.56619	0.05486	**0.00080**	0.0010	1.1113	0.2957
T4-Nearsoil	*Time*	2	0.32738	1.87699	0.04510	**0.00320**	0.0003	0.2050	0.8152
T4-Nearsoil	*Moisture*	1	0.25161	2.88516	0.03466	**0.00080**	0.0002	0.2241	0.6375
T4-Nearsoil	*Pore:Moisture*	1	0.19160	2.19708	0.02639	**0.00880**	-	-	-
T4-Farsoil	*Time*	2	0.34319	1.94229	0.05863	**0.00080**	0.0013	0.8474	0.4338
T4-Farsoil	***Pore***	1	0.30714	3.47648	0.05247	**0.00080**	0.0010	1.1832	0.2812
T4-Farsoil	*Plant*	2	0.29821	1.68774	0.05094	**0.00080**	0.0003	0.1571	0.8550
T4-Farsoil	*Moisture*	1	0.15576	1.76304	0.02661	**0.00960**	0.0000	0.0122	0.9124
T4-Farsoil	*Plant:Pore:Moisture*	1	0.14815	1.67689	0.02531	**0.01440**	-	-	-
T4-Control	*Time*	2	0.25182	1.49254	0.11267	**0.00240**	0.1105	4.4663	**0.0242**
T4-Control	***Pore***	1	0.19786	2.34541	0.08853	**0.00040**	0.1199	18.9037	**0.0003**
T4-Control	*Moisture*	1	0.16089	1.90716	0.07199	**0.00080**	0.0039	0.3283	0.5724

Time was treated as a fixed effect and included as a factor variable in the models (Time + Plant ^*^ Pore ^*^ Moisture, and Time + Pore ^*^ Moisture for just the control samples). The order of the factors was chosen to first remove variance from variables with the highest impact in the models and better investigate the remaining variables. Only factors with significant (P ≤ 0.05) Benjamini-Hochberg-corrected P-values are displayed. Factor explaining the highest R^2^ for each model and significant adjusted p-values are reported in bold.

The effect of time was the most important for the bacterial microbiome than the fungal microbiome, and significant pore:moisture and plant:pore:moisture interactions were also present in both fungal and bacterial communities, but only in T4 treatments, which represent the organic management type. Microbiomes clustered mainly according to plant and pore, as shown by the fungal (Figure 2) and bacterial (Figure 3) PCoA ordinations, as emphasized by 75% confidence ellipses.

### Alpha diversity

**Figure 4:**
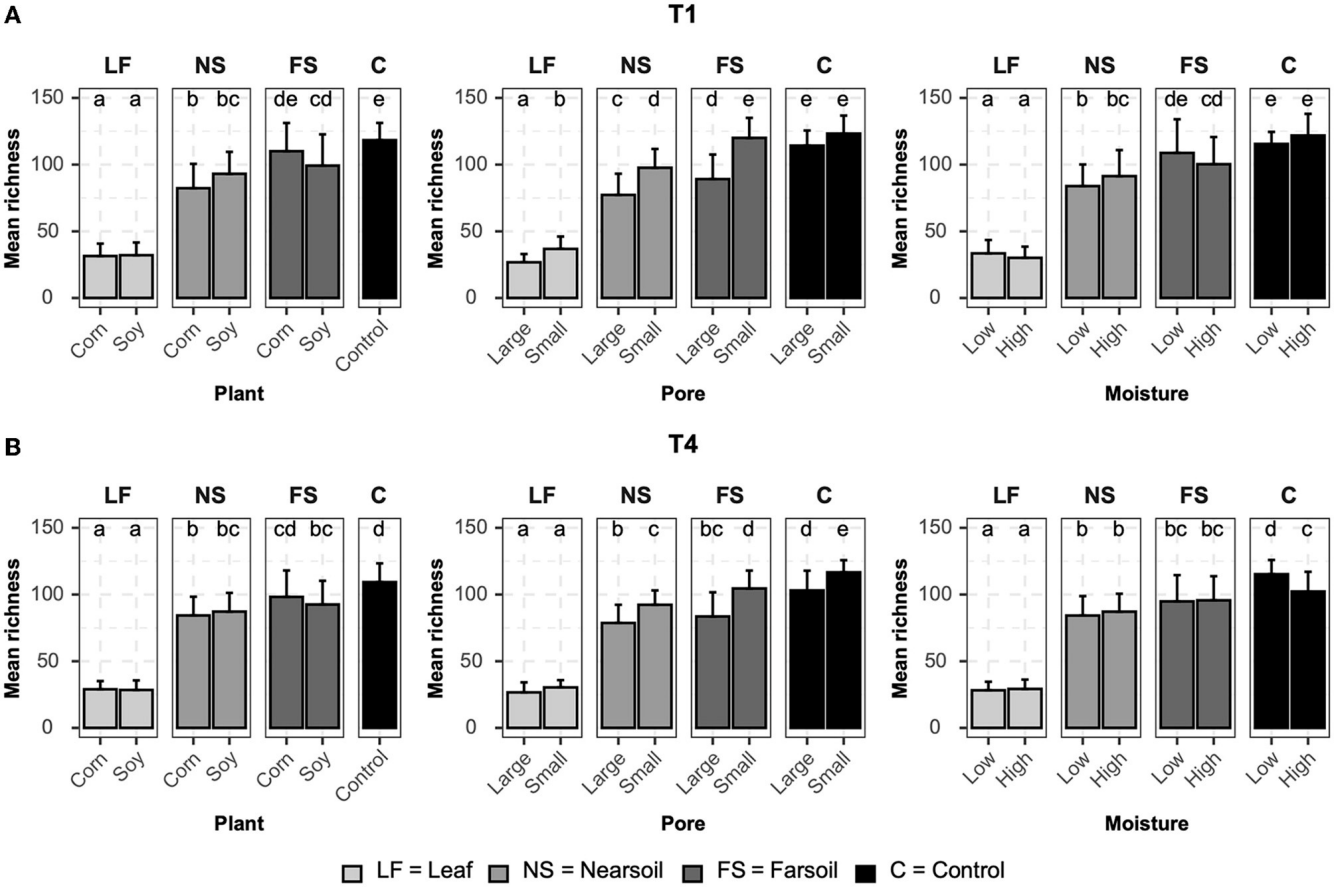
Bar plots showing significant differences (*P* ≤ 0.05 after Benjamini-Hochberg correction) in mean observed species richness (±standard deviation) for fungal microbiomes in **(A)** conventional (T1) and **(B)** organic (T4) managament samples. Samples were grouped according to treatment, and statistical differences were calculated across niche factors (leaf, near soil, far soil, and control) using pairwise Wilcoxon tests.

**Figure 5:**
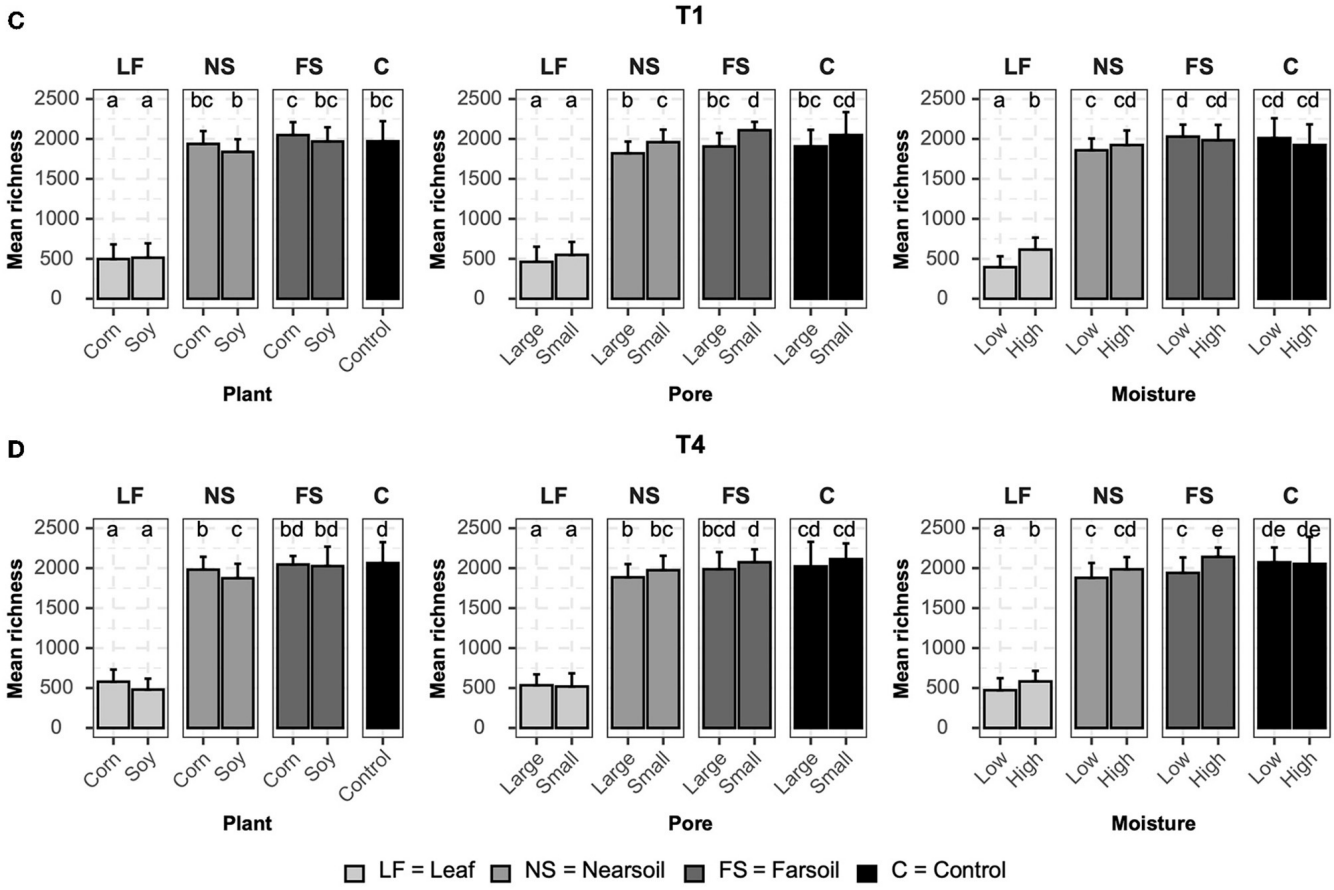
Bar plots showing significant differences (*P* ≤ 0.05 after the Benjamini-Hochberg correction method) in the mean observed species richness (±standard deviation) for bacterial microbiomes in **(A)** conventional (T1) and **(B)** organic (T4) managament samples. Samples were grouped according to treatment, and statistical differences were calculated across niche factors (leaf, near soil, far soil, and control) using pairwise Wilcoxon tests.

Alpha diversity measurements were also impacted primarily by niche, followed by other factors both in fungal (Figure 4) and bacterial (Figure 5) microbiomes. The bar plots also showed that there were no substantial alpha diversity differences between the T1 and T4 treatments. In particular, OTU richness in the leaf was considerably lower than that of the soils and controls, but differences between near soil, far soil, and control soil were also present. In the leaf niche, there was a significant (*P* ≤ 0.05 after BH correction) effect of pore size in the T1 sample of the fungal microbiome and a significant effect of moisture in both the T1 and T4 samples of the bacterial microbiomes. In the near soil niche, a higher richness was present in the small pores of T1 and T4 in fungi, but only in T1 in bacterial microbiomes. In the far soil niche, fungal microbiomes were affected by pore sizes in T1 and T4, but only in T1, a higher richness was detected for the bacterial microbiomes. The effect of pore sizes and moisture content was also significant in T1 and T4, respectively, but only in the fungal microbiome.

Regarding Shannon diversity, differences between niches were mostly limited to the leaves being different from soils and control samples. Additionally, Shannon diversity followed an opposite trend to richness, being higher in leaves (a more evenly abundant microbiome) and lower in soils, and this phenomenon was more evident in the bacterial (Supplementary Figure S3) than fungal (Supplementary Figure S4) microbiome. In particular, in the leaf niche, there was a significant (*P* ≤ 0.05 after BH correction) effect of moisture in both T1 and T4 in the fungal microbiome, but Shannon diversity was higher for the T4 bacterial microbiome in soy, small, and low moisture samples. Shannon diversity was significantly higher in the T1 near soil niche for the fungi but also in soy, large pores, and low moisture for the bacterial microbiome in T1 samples. No other significant differences were present. Large pores and low moisture samples were more even in T1 and T4 far soil samples of the bacterial microbiomes, respectively.

### Most abundant, variable, and significantly different OTUs across treatments

**Figure 6:**
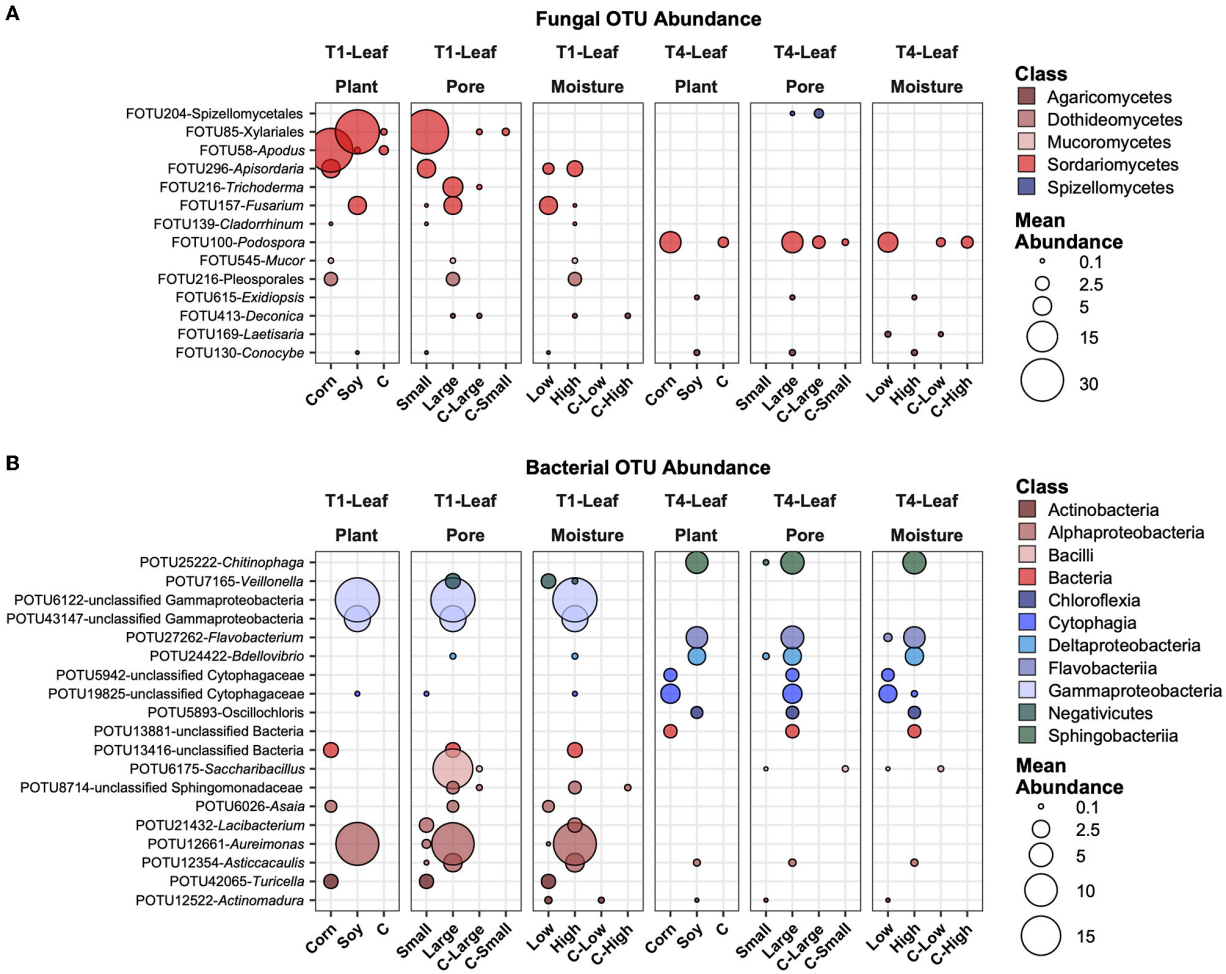
Top abundant, variable, and significantly different (pairwise Wilcoxon tests, *P* ≤ 0.05 after Benjamini-Hochberg correction method) OTUs across T1 and T4 treatments in the fungal **(A)** and bacterial **(B)** microbiomes of the leaf. Point sizes represent the mean abundance across samples at a treatment level. Only significantly different OTUs among the top 25 were selected among the most variable (OTUs with a difference in abundance between treatment levels ≥ the 50 percentile of the coefficient of variation for that OTU across samples for the fungi and ≥ the 75 percentile for the bacteria) and are shown.

In Figure 6, the most abundant fungal (Figure 6A) and bacterial (Figure 6B) OTUs are reported, averaged across all samples, and those that showed the highest variation and had significantly different mean abundance (*P* ≤ 0.05) after BH correction across the treatments. Some of these selected taxa showed different treatments. For example, FOTU85 (Xilariales) was higher in abundance in soy leaves and small pores, while present in small amounts in the control samples. FOTU58 (*Apodus* sp.) was instead significantly more abundant in corn leaves, but its abundance was not relevantly affected by other factors. Some OTUs were present and significantly different between factors in the in T1 treatment (e.g., FOTU545-*Mucor*), others only in the T4 treatment (e.g., FOTU100-*Podospora*). In the bacterial dataset, POTU25222-*Chitinophaga*, POTU27262-*Flavobacterium*, POTU24422-*Bdellovibrio*, and other unclassified bacteria were higher in soy samples with large pores and high moisture content in T1 samples. Other OTUs, such as unclassified Gammaproteobacteria, POTU6175-*Saccharibacillus*, and POTU12661-*Aureimonas*, were also higher in soy samples with large pores and high moisture content, but only in T1.

**Figure 7:**
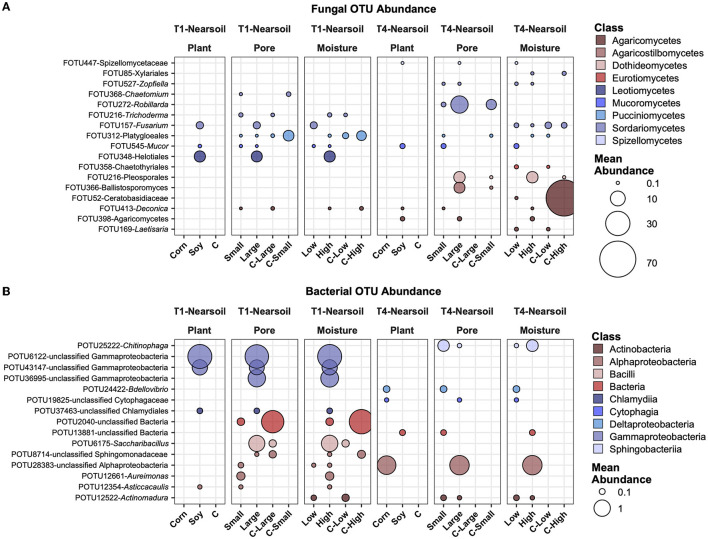
Top abundant, variable, and significantly different (pairwise Wilcoxon tests, *P* ≤ 0.05 after Benjamini-Hochberg correction method) OTUs across T1 and T4 treatments in the fungal **(A)** and bacterial **(B)** microbiomes of the Nearsoil. Point sizes represent the mean abundance across samples at a treatment level. Only significantly different OTUs among the top 25 were selected among the most variable (OTUs with differences in abundance between treatment levels ≥ the 50 percentile of the coefficient of variation for that OTU across samples for the fungi and ≥ the 75 percentile for the bacteria) and are shown.

In the near soil samples, FOTU272-*Robillarda* showed a higher abundance in large pores, together with FOTU216-Pleosporales and FOTU366-*Ballistosporomyces* (Figure 7A). Unclassified Gammaproteobacteria were higher in abundance in soy, large pores, and high moisture in T1, while an unclassified OTU in the Alphaproteobacteria was higher in T4, in corn, large pores, and high moisture samples. In T1, the most abundant and variable OTUs were associated with soil, while in the T4 treatment, they were associated with corn. POTU12661-*Aureimonas* and POTU12354-*Asticcacaulis* were the only two prokaryotes with a higher abundance in small soil pores (Figure 7B).

**Figure 8:**
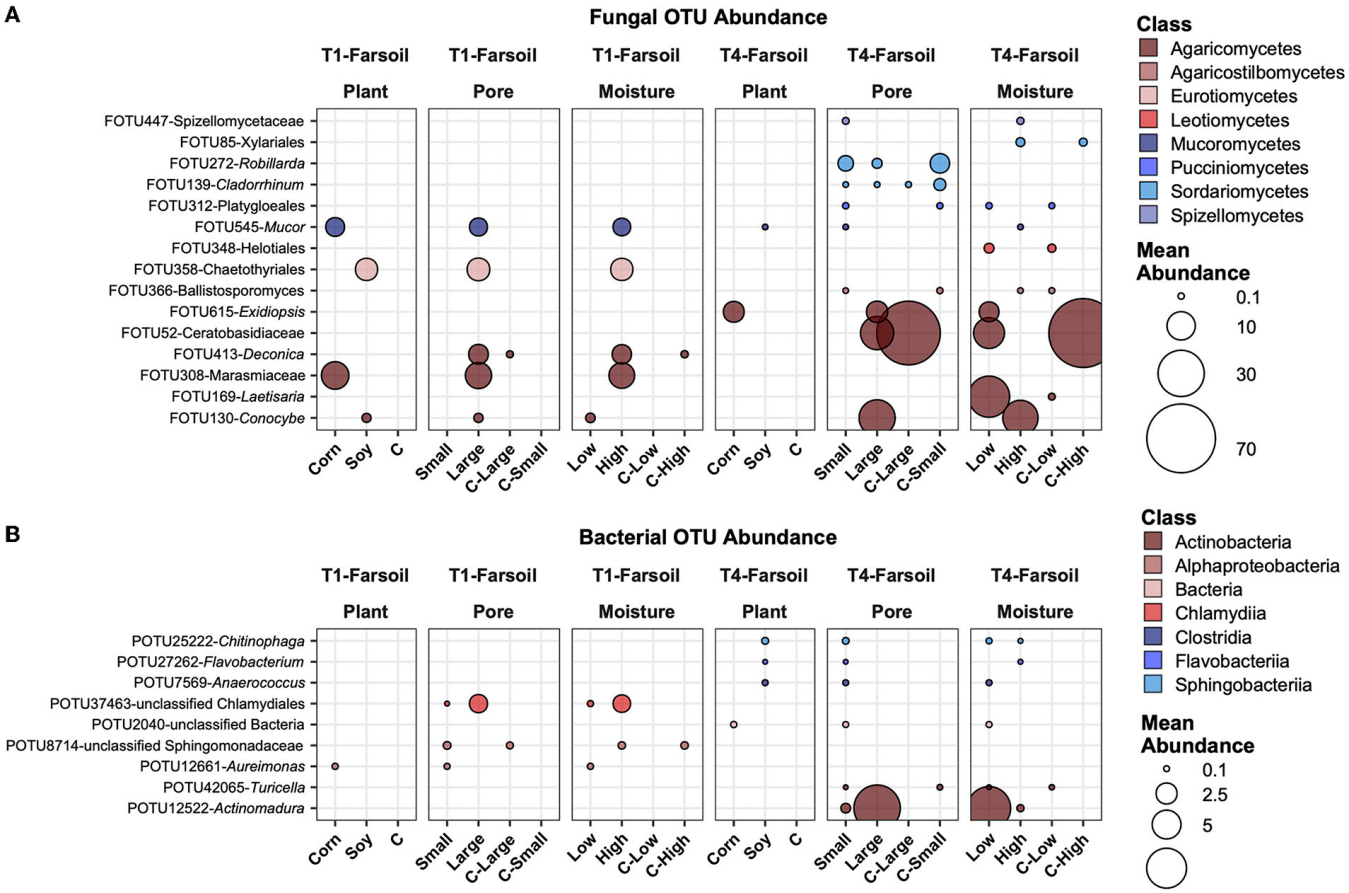
Top abundant, variable, and significantly different (pairwise Wilcoxon tests, *P* ≤ 0.05 after Benjamini-Hochberg correction method) OTUs across T1 and T4 treatments in the fungal **(A)** and bacterial **(B)** microbiomes of the far soil. Point sizes represent the mean abundance across samples at a treatment level. Only significantly different OTUs among the top 25 were selected among the most variable (OTUs with a difference in abundance between treatment levels ≥ the 50 percentile of the coefficient of variation for that OTU across samples for the fungi and ≥ the 75 percentile for the bacteria) and are shown.

In the far soil samples, some of the same OTUs present in the near soil samples were abundant and variable across groups. For example, FOTU545-*Mucor* (Figure 8A) was shown to be higher in corn with large pores and high moisture content, while FOTU358-Chaetothyriales (Figure 8B) was higher in soy with large pores and high moisture content; both were higher in T1 compared to T4, highlighting inherent differences between soil communities. In general, in T4, the most abundant OTUs that vary across factors were also present in the control samples, while in T1, they were absent.

## Discussion

In this study, we tested the impact of the prevalence of soil pores of a certain size range, different soil moisture contents, and decomposition of plant tissue of different qualities on fungal and bacterial dynamics across space and time. We explored both the tissue itself and the surrounding detritusphere. We found that soils subjected to long-term differences in soil management practice, i.e., conventional vs. organic management, had the greatest influence on microbial community structure, likely the result of differences in plant diversity but also due to increases in soil organic matter in the organic management in these field soils (Syswerda et al., 2011). That is in agreement with observations by Epp Schmidt et al. (2022) on the soils from similar management practices, also after extended implementation. The microbial community in the long-term biologically based treatment showed greater microbial richness (Figure 3), consistent with a number of past reports (de Graaff et al., 2019).

As expected, the greatest contrast in microbial community composition was observed between the community on the decomposing residue and the communities of the surrounding soil. Leaf microbiomes were less species rich as compared to the adjacent soil (Figure 3), likely reflecting the special environment dominated by leaf decomposers, organisms that benefited from their necromass, and predators. The decomposing leaves, providing a carbon and nutrient supply, drive microbial functioning in the soil microenvironment (Figure 3).

As we reported in a companion study, decomposition rates and magnitudes substantially differed between microcosms with incubated corn vs. soybean leaves (Kravchenko et al., 2017). While >85% of the soybean residue was completely decomposed after 7 days of incubation, only 30–50% of the corn residue was decomposed by that time. However, surprisingly, the effect of plant species on the composition of the microbial community was relatively minor, especially for bacteria. Mortierellomycetes, a clade of fungi reportedly abundant in agricultural conventionally and organically managed systems (Epp Schmidt et al., 2022; Benucci et al., 2023) and known to include soil saprotrophs as well as plant growth promoters (Põlme et al., 2020; Vandepol et al., 2022), had a greater abundance on soybean leaves than corn leaves. Agaricomycetes and Sordariomycetes groups, known to include large proportions of wood and litter saprotrophs (Põlme et al., 2020), were more abundant in corn than in soybean leaves.

We hypothesized that micro-environments within small pore-dominated soil, especially when accompanied by low soil moisture, would stimulate greater diversity of microbial communities. Smaller and less hydraulically connected pore spaces generate more fragmented microhabitats, shielding inhabitants from predation and competition (Tiedje et al., 2001; Wolf et al., 2013; Bickel and Or, 2020). This effect was expected to be more pronounced in bacteria than in fungi since hyphal growth was assumed to enable fungi to easily navigate and spread through the pore space, allowing them greater resistance to fluctuations in local environmental conditions (Barnard et al., 2013; Nunan et al., 2020). Our findings only partially supported this hypothesis (Figure 3). Greater bacterial diversity was indeed observed in small pore treatments than in large pore treatments, but it was statistically significant only in the soil of conventional agriculture and was only a numeric trend in organic management. However, a greater diversity of fungi was consistently observed in the soils of both management practices. The result suggests a greater than expected sensitivity of fungi to micro-environmental conditions, even at a few-cm spatial scale.

However, the association between soil moisture and microbial richness was either absent or the opposite of what we anticipated. Moisture did not influence fungal richness, and on decomposing residues and partially in the surrounding soil, greater richness was associated with higher moisture (Figure 3). It is possible that the lower moisture of the study limited many organisms and selected for those tolerant of drier conditions, while the optimal (field capacity) moisture of our high soil moisture treatment provided an optimal growth environment. Indeed, the moisture corresponding to field capacity was reported as beneficial to bacterial diversity in both experimental works (Carson et al., 2010) and theoretical considerations (Bickel and Or, 2020).

Nevertheless, for several bacterial groups, the associations with pores were consistent with the associations with soil moisture levels, suggesting the contribution of general micro-environmental effects to the performance of these microorganisms during the experiment. Betaproteobacteria and Bacteriodetes were in greater abundance in both large-pore soils and at higher soil moisture (Figure 5). Gammaproteobacteria and Shingobacteria were in greater abundance in both small-pore soils and at lower soil moisture. Actinobacteria, a phylum known to be resistant to desiccation (Bardgett and Caruso, 2020), was also notably more abundant in both small-pore soils and at lower soil moisture. A number of Acidobacteria groups, which are usually described as oligotrophs resistant to harsh environments, were also in greater abundance in small pores, as were Anaerolinea. The higher abundance of Acidobacteria in the small pore treatment, with its lower oxygen supply, was expected. Consistent with our findings, Xia et al. (2022) reported a greater abundance of Actinobacteria in smaller pores and drier conditions and a greater abundance of Betaproteobacteria in large pores.

## Conclusion

We reported here that decomposing leaves in the soil drive microbial activity and turnover over time. As we hypothesized, incubated fresh plant detritus was shown to harbor a reduced diversity but a more even microbiome composition compared to that of the adjacent communities in the soil and was the most important factor explaining fungal and bacterial microbiomes across space. We did not entirely expect to have such an important effect of management type (organic vs. conventional), which was also variable across the different niches, and impactful on the effect of pore size on both bacterial microbiomes. Soil pores and moisture content were influenced by both niche and management type and were important in shaping bacterial communities, which are known to rely on water films for dispersal and were more dynamic over time compared to fungi. In contrast, and as hypothesized, plant species had a greater effect on the fungal community composition over time. Together, these results contribute to our understanding of the decomposition of carbon residues in the soil and the factors that regulate the microbes that drive soil C and nutrient cycling.

## Data availability statement

Raw ITS and 16S sequences .fastq reads were submitted to the Sequence Read Archive (Leinonen et al., 2011) under the bioproject number PRJNA938072. Data sets, and R code developed to analyze them, are accessible at: https://github.com/Gian77/Scientific-Papers-R-Code. Additional material associated with this article is also available in the Supplementary material.

## Author contributions

GBe: performed the data analysis, created figures and tables, and wrote the manuscript. ET: conceived experimental approaches and carried out the research experiment and sampling. FY: processed samples and generated amplicon sequence data. TM: analyzed data and created figures and tables. GBo: wrote and edited the manuscript. AK: conceived experimental approaches and wrote and edited the manuscript. All authors contributed to the article and approved the submitted version.
