# Supplementary material for: The microbiome structure of decomposing plant leaves in soil depends on plant species, soil pore sizes, and soil moisture content
Source: Front Microbiol. 2023 Aug 14;14:1172862. doi: 10.3389/fmicb.2023.1172862 (PMC10461183; doi:10.3389/fmicb.2023.1172862)
Supplement: Supplementary file 2 [file Image_2.pdf]

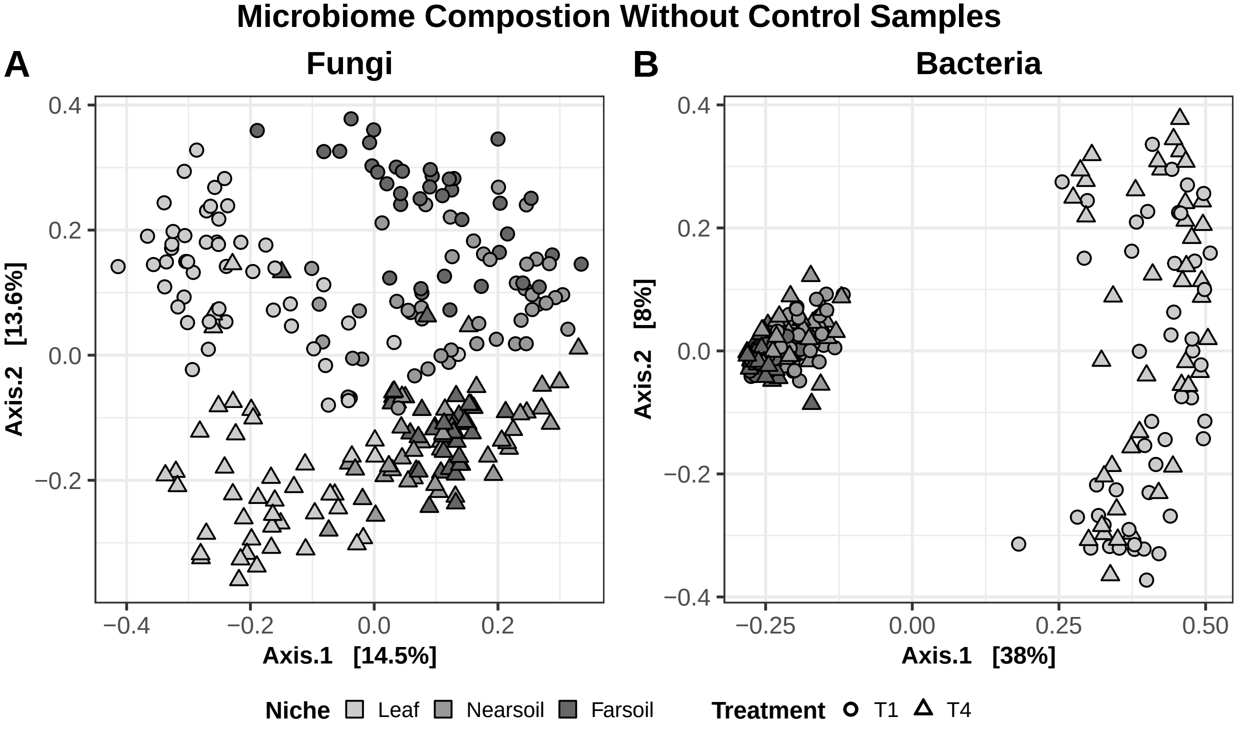
Microbiome Compostion Without Control Samples
A
Fungi
B
Bacteria
0.4
0.2
0.0
−0.2
Axis.2 [13.6%]
−0.4
−0.2
0.0
0.2
Axis.1 [14.5%]
0.4
0.2
0.0
−0.2
−0.4
Axis.2 [8%]
−0.25
0.00
0.25
0.50
Axis.1 [38%]
Niche
Leaf
Nearsoil
Farsoil
Treatment
T1
T4
