# Supplementary figures and images for: The microbiome structure of decomposing plant leaves in soil depends on plant species, soil pore sizes, and soil moisture content
Source: Front Microbiol. 2023 Aug 14;14:1172862. doi: 10.3389/fmicb.2023.1172862 (PMC10461183; doi:10.3389/fmicb.2023.1172862)

# Bacterial Shannon Index

**A**

**T1**

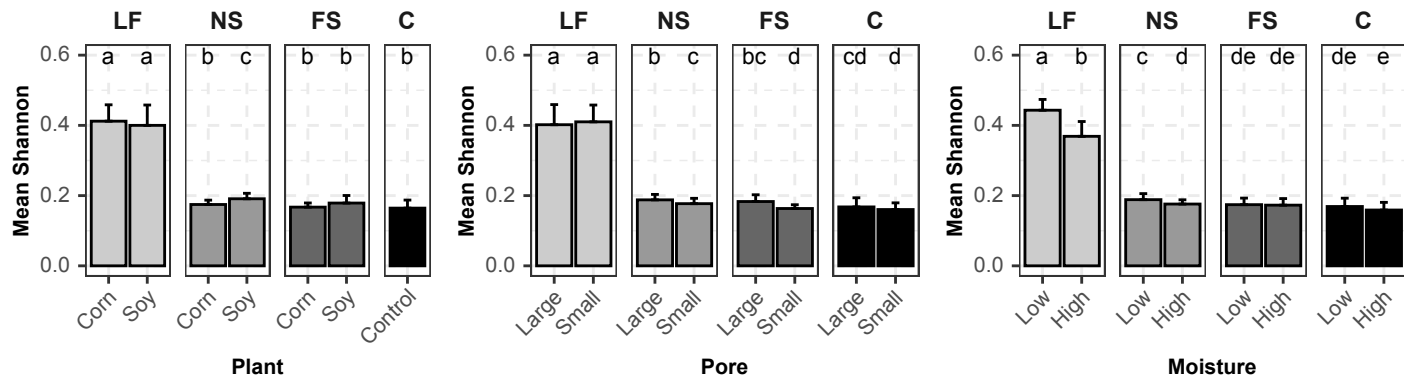

**B**

**T4**

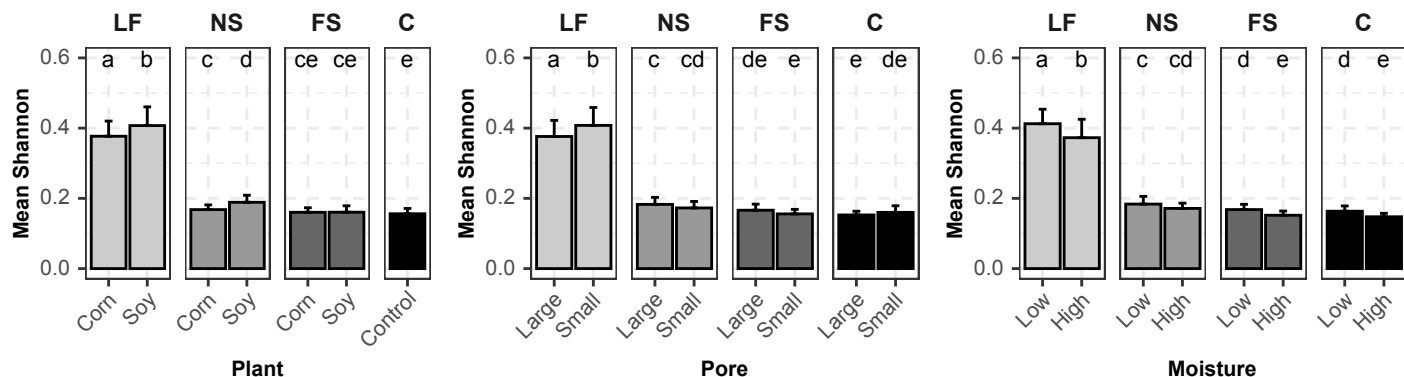

LF = Leaf 
  NS = Nearsoil 
  FS = Farsoil 
  C = Control

Supplement: Supplementary file 3 [file Image_3.pdf]

# Fungal Shannon Index

**A**

**T1**

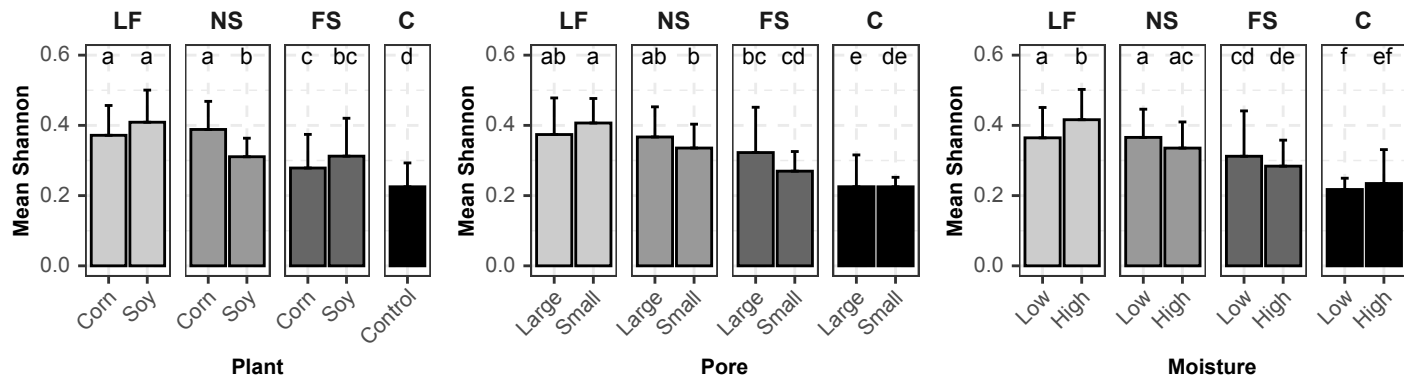

**B**

**T4**

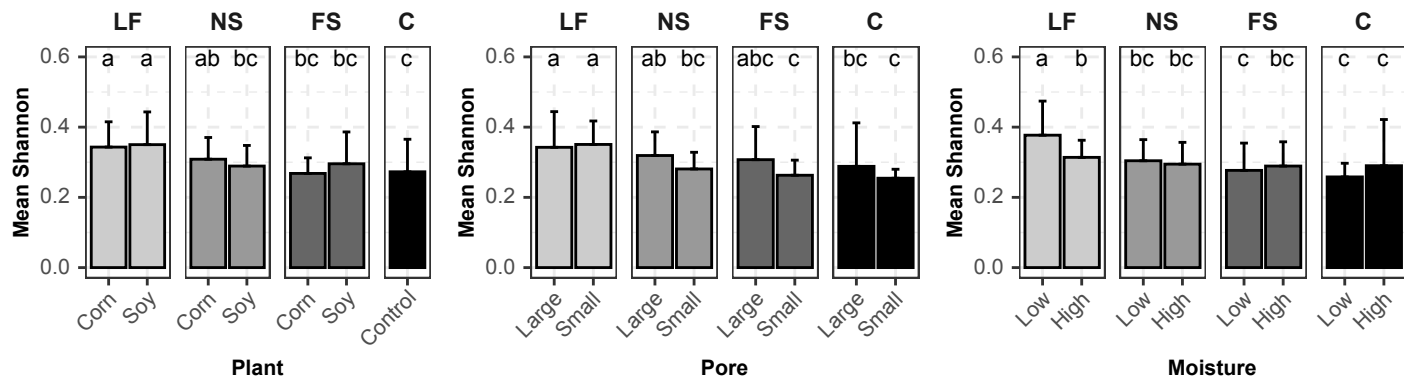

LF = Leaf 
  NS = Nearsoil 
  FS = Farsoil 
  C = Control

Supplement: Supplementary file 4 [file Image_4.pdf]
