# Supplementary material for: The microbiome structure of decomposing plant leaves in soil depends on plant species, soil pore sizes, and soil moisture content
Source: Front Microbiol. 2023 Aug 14;14:1172862. doi: 10.3389/fmicb.2023.1172862 (PMC10461183; doi:10.3389/fmicb.2023.1172862)
Supplement: Supplementary file 5 [file Table_1.pdf]

		Permanova				*Betadisper*		
**Fungi**	**Df**	**Sum.Sq**	**F**	**R^2**	**P.adj**	**Sum.Sq**	**F**	**P.adj**
Pore	1	0.1057	1.0322	0.0623	0.3606	0.2160	40.0528	**0.0001**
Treatment	1	0.7000	6.8328	0.4122	**0.0016**	0.0332	3.9210	0.0486
Pore:Treatment	1	0.0730	0.7126	0.0430	0.6566	-	-	-
**Bacteria**								
Pore	1	0.1030	1.1592	0.0973	0.1591	0.0069	0.2548	0.6115
Treatment	1	0.1704	1.9181	0.1610	**0.0010**	0.0184	0.6662	0.4114
Pore:Treatment	1	0.0743	0.8360	0.0702	0.8808	-	-	-
