# Supplementary material for: The microbiome structure of decomposing plant leaves in soil depends on plant species, soil pore sizes, and soil moisture content
Source: Front Microbiol. 2023 Aug 14;14:1172862. doi: 10.3389/fmicb.2023.1172862 (PMC10461183; doi:10.3389/fmicb.2023.1172862)
Supplement: Supplementary file 6 [file Table_2.pdf]

	Permanova					Betadisper		
**Fungi**	**Df**	**Sum.Sq**	**F**	**R^2**	**P.adj**	**Sum.Sq**	**F**	**P.adj**
Slice	2	9.619	36.310	0.160	**0.003**	0.0848	6.7032	**0.0015**
Treatment	1	6.534	49.327	0.109	**0.003**	0.0112	1.5327	0.2169
Plant	1	2.343	17.688	0.039	**0.003**	0.0014	0.2361	0.6275
Pore	1	1.605	12.115	0.027	**0.003**	0.1472	28.5655	**0.0000**
Moisture	1	0.636	4.802	0.011	**0.003**	0.0108	2.0232	0.1562
Slice:Plant	2	2.201	8.307	0.037	**0.003**	-	-	-
Slice:Treatment	2	1.709	6.451	0.028	**0.003**	-	-	-
Slice:Moisture	2	0.912	3.445	0.015	**0.003**	-	-	-
Slice:Pore	2	0.863	3.257	0.014	**0.003**	-	-	-
Treatment:Pore	1	0.533	4.023	0.009	**0.003**	-	-	-
Treatment:Plant:Pore:Moisture	1	0.436	3.292	0.007	**0.003**	-	-	-
**Bacteria**								
Slice	2	24.212	122.342	0.387	**0.003**	1.3344	428.8072	**0.0000**
Pore	1	1.619	16.357	0.026	**0.003**	0.0080	0.3284	0.5671
Time	2	1.482	7.499	0.024	**0.003**	0.00408	0.085069	0.9185
Moisture	1	1.339	13.535	0.021	**0.003**	0.0010	0.0431	0.8357
Treatment	1	1.282	12.951	0.021	**0.003**	0.0076	0.3064	0.5804
Plant	1	0.885	8.944	0.014	**0.003**	0.0129	0.5573	0.4560
Slice:Moisture	2	1.592	8.046	0.025	**0.003**	-	-	-
Slice:Pore	2	1.580	7.984	0.025	**0.003**	-	-	-
Slice:Treatment	2	1.300	6.569	0.021	**0.003**	-	-	-
Slice:Plant	2	1.143	5.778	0.018	**0.003**	-	-	-
Treatment:Pore	1	0.437	4.419	0.007	**0.042**	-	-	-
